# Analytic Markovian Rates for Generalized Protein Structure Evolution

**DOI:** 10.1371/journal.pone.0034228

**Published:** 2012-05-23

**Authors:** Ivan Coluzza, James T. MacDonald, Michael I. Sadowski, William R. Taylor, Richard A. Goldstein

**Affiliations:** 1 Department of Physics, University of Vienna, Vienna, Austria; 2 Division of Molecular Biosciences, Imperial College London, London, United Kingdom and Centre for Synthetic Biology and Innovation, Imperial College London, London, United Kingdom; 3 National Institute for Medical Research, The Ridgeway, London, United Kingdom; University of Rome, Italy

## Abstract

A general understanding of the complex phenomenon of protein evolution requires the accurate description of the constraints that define the sub-space of proteins with mutations that do not appreciably reduce the fitness of the organism. Such constraints can have multiple origins, in this work we present a model for constrained evolutionary trajectories represented by a Markovian process throughout a set of protein-like structures artificially constructed to be topological intermediates between the structure of two natural occurring proteins. The number and type of intermediate steps defines how constrained the total evolutionary process is. By using a coarse-grained representation for the protein structures, we derive an analytic formulation of the transition rates between each of the intermediate structures. The results indicate that compact structures with a high number of hydrogen bonds are more probable and have a higher likelihood to arise during evolution. Knowledge of the transition rates allows for the study of complex evolutionary pathways represented by trajectories through a set of intermediate structures.

## Introduction

Protein sequence evolution occurs at the genetic level with a rate that varies from protein to protein, depending upon several factors such as the processing of the protein in the cell (e.g. translation time) [1], [2], or molecular characteristics specific to each protein [3]–[5], as well as from interactions with other proteins (reviewed in Pal et al [6]). In contrast, the nature and rate of protein structural evolution is much less well understood. Viksna et al. [7] presented an estimate of the rate of structural changes based on the measure of topological distances between proteins structures. Meyerguz et al. [8] grouped all known proteins into basins corresponding to the common native structures. From the collected data the authors have then built a network of sequences and considered the frequency of “transition" sequences (separated by a single point mutation from a different basin). Structural evolution has also been studied in the context of lattice protein model by Deeds et al. [9], where the structural similarities among all possible 103346 distinct structures of a 3×3×3 lattice polymer have been mapped. Other work has concentrated on structural topologies connected by a relatively small set of structural evolutionary moves (e.g domain swapping, or duplications) [3], [5], [10], [11].

In what follows we will introduce a novel theoretical framework for the characterization of the evolution process between two target structures that, instead of considering only proteins present in the Protein Data Bank (PDB) [12], is based on an arbitrary set of structures constructed via a realistic off-lattice coarse-grained model. If for a moment we consider the entire evolutionary process without focusing on a detailed description of the cell physiology, then the evolutionary process is equivalent to screening a large number of different sequences under the constraint that only few structures are acceptable. The total evolutionary path can then be represented as a sequence of transitions between the allowed structures (stepping stones). Such stepping stones represent the possible structures that are still allowed by the selection function and are not identical to the initial and final target structure. The number of intermediate structures reflects the degree of restriction applied to the evolutionary process, hence the larger the number of stepping stones the more closely the evolutionary process approximates a free drift in protein space. The total evolutionary trajectory between two targets is then represented as a path connecting the stepping stones, where each jump is weighted by its probability of occurrence. Accordingly the main objective of our work is to measure the rate of each elementary jump and identify the analytic dependence of such rates from a small set of structural differences. Similarly to the recent work of Lobkovsky et al. [4], we associated to each structure a set of sequences, or “islands", that can fold into the respective configuration. Each jump should then only consider trajectories between the islands without considering intermediate configurations. In other words, we need to sample the sequences that fold into each stepping stone and then define the evolutionary rates between the resulting islands. For this purpose, we will use the “Caterpillar" coarse-grained protein model recently introduced by Coluzza [13]. In contrast to the model used by Lobkovsky, the Caterpillar model was able to refold designed sequences into protein structures taken from the PDB with a very high accuracy. Moreover the designed sequences had large similarities with the corresponding wild type sequences, to the point that the model was able to refold a wild type protein to its native structure with remarkable accuracy. Hence, the Caterpillar model is an ideal tool to study the the restriction imposed on the sequence space by the constraints of protein structure space because it is both fast and reproduces protein-like structure and sequence features.

In what follows we will first describe the model used to represent the proteins, the method used to construct the intermediate stepping stones between the target structures, the protein design method and the theory used to calculate the single jumping rates. Finally, we will present the results with the analytic dependence of the elementary evolutionary rates on a small set of physical parameters, namely the difference in the number of hydrogen bonds and in the number of total contacts between the residues. This approach will not be able to predict the evolutionary process protein by protein but hopefully will highlight the universal dependence of the total evolutionary rate on physically measurable quantities such as the number of locally available structures (the fewer structures the stronger the constraint) and their spread in terms of the distribution of jumping probabilities (the larger the distance the stronger the constraint).

## Materials and Methods

### Generation of the stepping stones

**Figure 1:**
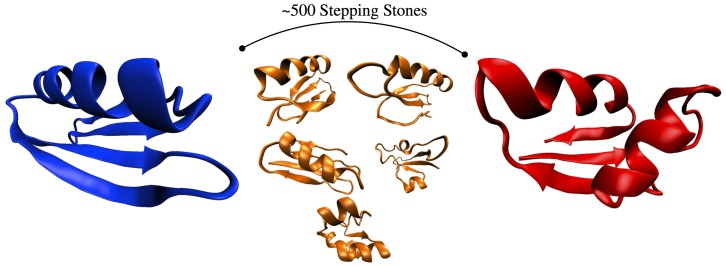
Schematic representation of the evolutionary process between the protein Immunoglobulin Binding Protein (1PGB) in blue on the left and the chain X of the 50's subunit of a secm-stalled E. Coli ribosome complex (2GYC) in red on the right. Between the two proteins we show a few of the 500 stepping stones generated with our procedure.

We consider two naturally occurring protein structures that represent the endpoints of the evolutionary process. We chose two proteins of equal length with substantial structural difference, for this purpose we used the Immunoglobulin Binding Protein (1PGB) and the chain X of the 50S subunit of a secm-stalled E. Coli ribosome complex (2GYC) (see Fig. 1). The secondary structure of the two proteins is represented by a string where each letter corresponds to a residue and the type of letter indicates if the amino acids is part of a helix (H), strand (E) or other (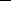). Thus for the protein 1PGB we have: 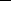
EEEEEEE
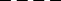
EEEEEEE
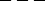
HHHHHHHHHHHHHHH
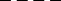
EEEEE
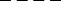
EEEEE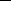 (a pattern that we will refer to as 1PGB-E1 1PGB-E2 1PGB-H1 1PGB-E3 1PGB-E4), while 2GYC can be represented by: EEEEE
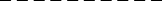
HHHHHHHH
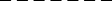
EEEE
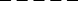
HHHHHHH
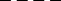
EE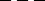 (which we will refer to as 2GYC-E1 2GYC-H1 2GYC-E2 2GYC-H2 2GYC-E3).

In order to generate the stepping stones we constructed intermediate sequences of secondary structural states according to the following rules: (i) Secondary structure elements can be added, deleted, shortened, or lengthened (ii) Secondary structure elements can be lengthened by converting an adjacent ‘
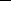
’ to an ‘E’ or ‘H’. (iii) Secondary structure elements can be shortened by converting an ending ‘E’ or ‘H’ to an ‘
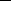
’. (iv) Helices must be between 4 and 15 in length. Strands between 2 and 8 in length. (v) Helices can be added by converting a 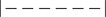 to a ‘
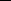
HHHH
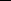
’. Strands can be added by converting a 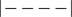 to a ‘
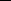
EE
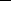
’. (vi) Helices can be deleted by converting a 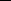
HHHH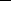 to a 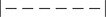. Strands can be deleted by converting a ‘
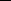
EE
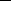
’ to a 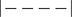. (vii) There must always be at least one unstructured 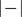 between secondary structure elements. (viii) The number of Es and Hs must remain between 20 and 45. (ix) The move must change the current structure so that it more closely matches the final structure.

7 paths of intermediate secondary structure profiles were created, representing the different parsimonious ways the secondary structure of 1PGB could be converted to 2GYC using the previously described rules. Each path was approximately 50 steps in length. A brief description of the pathways follows:

1. the disappearance of the three central secondary structure elements (1PGB-E2, 1PGB-H1, and 1PGB-E3) and their replacement by three new secondary structures (2GYC-H1, 2GYC-E2, 2GYC-H2),
2. the disappearance of 1PGB-E2, the movement of 1PGB-H1 and 1PGB-E3 to form 2GYC-H1 and 2GYC-E2, and the creation of 2GYC-H2,
3. the disappearance of 1PGB-E2 and 1PGB-E3, the movement of 1PGB-H1 to form 2GYC-H1, and the creation of 2GYC-E2 and 2GYC-H2,
4. the disappearance of 1PGB-E2 and 1PGB-H1, the movement of 1PGB-E3 to form 2GYC-E2, and the creation of 2GYC-H1 and 2GYC-H2,
5. the disappearance of 1PGB-E3, the movement of 1PGB-E2 and 1PGB-H1 to form 2GYC-E2 and 2GYC-H2, and the creation of 2GYC-H1,
6. the disappearance of 1PGB-E2 and 1PGB-E3, the movement of 1PGB-H1 to form 2GYC-H2, and the creation of 2GYC-H1 and 2GYC-E2,
7. the disappearance of 1PGB-H1 and 1PGB-E3, the movement of 1PGB-E2 to form 2GYC-E2, and the creation of 2GYC-H1 and 2GYC-H2.

In all cases the terminal beta strands maintained their identities.

**Figure 2:**
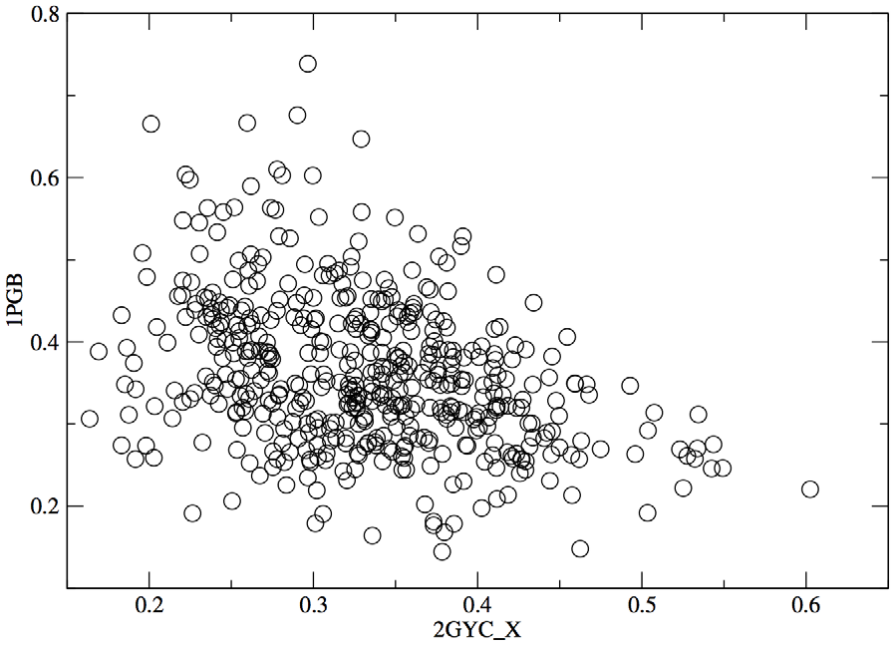
Plot of the distribution of stepping stones as a function of the percentage of native like contact (for a 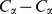 distance below 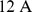) that each structures have in common with either 1PGB or 2GYC.

The second step consisted of combinatorially generating the idealised structural 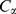 models with folds compatible with the intermediate secondary structure strings and the “rules" of protein topology (e.g. right-handed beta-alpha-beta connections, no crossing loops, etc.) [14]. This step resulted in around 45,000 structures with 490 distinct fold topologies. A representative model for each distinct topology was selected and refined into full backbone models using the procedure from MacDonald et al. [15]. The result was a list of 490 structures, plotted in figure (Fig. 2) by percentage contacts (where a contact is is counted for any 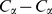 distance below 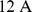) in common with 1PGB and 2GYC to show how much of the configurational space separating the two end points is covered by the stepping stones.

The stepping stone structures were used as targets for sequence design to generate sequence “islands", each containing a large number of sequences folding into the appropriate structure. We describe the design method and the theory that defines the jumping rates between the islands below.

### Sequence Design

There are several ways to design the protein sequences to fold into a specific backbone conformation. We reported one such strategy in ref. [16]. In what follows we use a novel version of this method [13]. The general principle remains unchanged: sequences are generated by minimizing the energy of the target configuration(s) and, at the same time, by maximizing the number of possible letter permutations to increase the sequence heterogeneity. In the present study we use this scheme to generate the population of sequences that belong to each island. Once the best sequences are chosen according to our design scheme, we can proceed to test if the desired folding properties have been achieved by folding the sequence to see if it attains the target structure. Owing to limitations of computational time this test step was only applied to a small selection of stepping stones and for the starting and the end structures.

### Design Algorithm

In order to obtain well folding proteins for a given stepping stone we applied the method developed by Coluzza [13] that has proved to be very effective for the design and refolding of caterpillar proteins. The basic design moves are single point mutations. We compute the difference 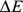 between the energy of the native state for the new sequence compared with the pre-mutation sequence. As in the conventional Metropolis scheme, the acceptance of trial moves depends on the ratio of the Boltzmann weights at temperature 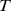 of the new and old states. However, if this were the only criterion there would be a tendency to generate homo-polymer chains with a low energy, rather than chains that fold selectively into the desired target structure. To ensure the necessary heterogeneity, we impose the following acceptance criterion
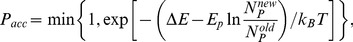
(1)where 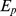 is an arbitrary parameter that plays the role of an energy scaling factor, and 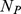 is the number of permutations that are possible for a given set of amino acids. 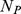 is given by the multinomial expression
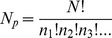
(2)where 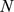 is the total number of monomers and 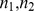 etc are the number of amino acids of type 1,2,. While sampling the sequence space with a Monte Carlo scheme, we fix the energy scale factor 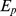 at high values. In doing so we generate an heterogeneous composition of amino acids. In contrast to previous work [4], we used a 20 letter alphabet since this helps to reduce the degeneracy of the ground state and so mimic the folding behavior of a real system. During a Monte Carlo run of several million cycles, a large number of distinct sequences are generated (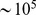). In order to increase the sampling we have applied the Virtual Move Parallel Tempering (VMPT) [17] sampling method, running the simulation at several design temperatures, 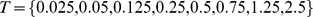. Good sequences are expected to be found at lower temperature, much as proteins have folding temperatures below which they are folded. We then computed the Shannon entropy per residue 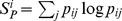 for protein G, where 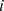 is the residue and 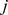 is the residue type, and we compared the distribution of values of 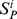 calculated over a set designed sequences with the distribution obtained for the the natural population (PF01053 [18]), for various design temperatures.

**Figure 3:**
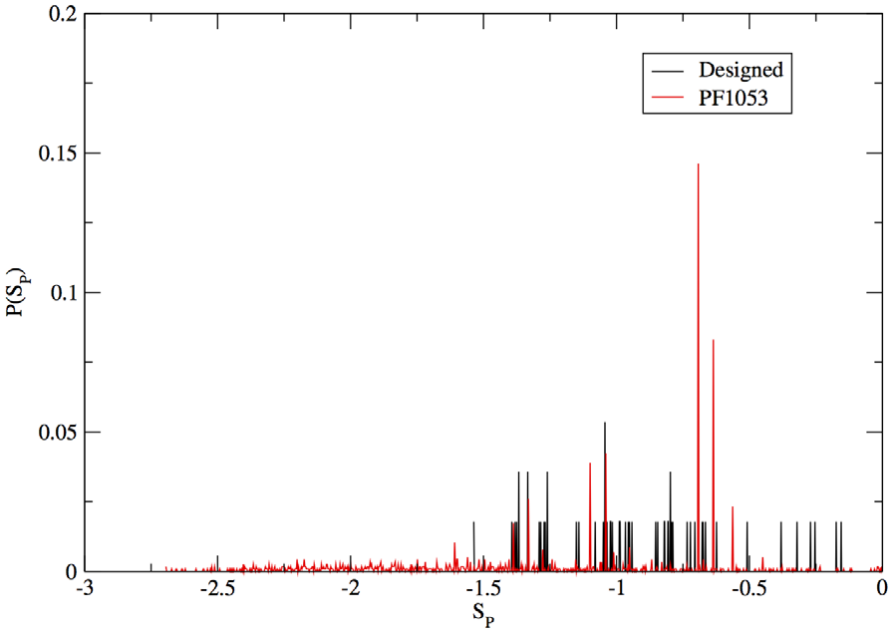
Plot of the distribution of the site Shannon entropy 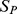 calculated for the family of natural sequences of 1PGB (PF01053) in red and for the designed sequences in black.

We found (see Fig. 3) that the sequence populations generated for protein G at the design temperature 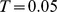 had the closest variability compared to the corresponding family of natural sequences. Using the scheme just described we performed a design simulation for every stepping stone and hence generated a distribution of folding sequences for each of them. From now on we will refer to the stepping stones with their corresponding distribution of folding sequences as “islands". With the islands in hand a method is now required to characterize the probability of transition between islands. This method is described in the following section.

### Transition Rate Calculations

The objective is to sample the rate at which an ensemble of sequences defined by the design procedure with target structure 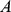 will evolve to an equivalent ensemble defined by the design of structure 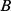. The first thing we observed is that the overlap between the most probable sequences of 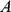 and of 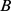 is very small, independently of the structural differences between 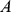 and 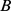. In other words provided that the structures are not identical the Hamming distance between the ensemble of folding sequences is always large and is of the same order of magnitude as the spread measurable in the distribution of sequences that fold into either target (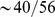 residues). If the distributions were represented as spheres in the N-dimensional space of sequences [19]–[22], then the design data indicate that where the distributions interpenetrate the overlap volume is very small. This does not necessarily mean that the evolutionary process must proceed with very large jumps with many concurrent mutations, but it means that the folding sequences that are in “common" (so with small Hamming distance) between the two distributions are quite rare, hence the evolutionary rate is highly dependent on the probability of finding such sequences that are still able to fold but are separated by a small number of mutations. For this reason we will assume that neutral evolution inside each island occurs at a higher rate than it does between islands. It is important to stress that with neutral evolution we do not mean that all sequences in the island have the same probability of folding into the target structure, but that such probability is higher than a threshold which we will define below. Hence the jumping rate from the island associated with structure 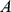 to 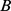 is going to be equal to the rate of accumulating enough mutations for each sequence of the island of 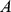 to become equal to one of sequences in the island of 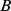, as the evolutionary process will spontaneously continue towards the optimal sequences of 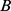 at a much faster rate.

In order to measure such a rate we will define a quantity called the “committor" that is a measure of the status of the evolutionary process. Once the evolutionary process starting from 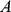 reaches a certain threshold value of the committor we say that that trajectory is now committed to 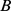 or it will spontaneously reach 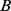. An exact measure of this quantity would involve an extensive sampling of all possible mutation trajectories that start in each sequences of 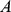 and end in 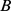. However this study would involve too many resources for the number of islands and the population size that we intend to treat. Instead we will use a natural definition of the committor for this problem, and we will define it as the point at which a sequence goes from having lower total energy in structure 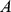 to having lower energy in 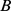. This choice can be justified as a measure of the propensity of that sequences to fold into 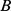 instead of 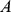 because if we assume that the entropic contribution to the free energy of the native structure is the same across all stepping stones, then the only relevant pressure is the energetic contribution. The probability of observing such a sequence can then be measured using the Boltzmann distribution function in the space of all possible proteins (all sequences on all structures).

Once these states are reached we define the trajectory as committed to evolve towards 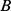, at which point evolution proceeds spontaneously with a speed that depends on the mutation rate 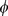 which is assumed to be equal among all the evolutionary processes. The rate of jumping from 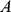 to 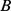 is
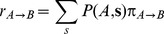
(3)where 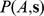 is the probability of finding the system in a sequence 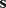 and structure 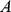 (same for 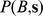), and 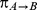 is the transition function that we define:
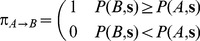
(4)Hence when 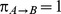 then the evolutionary trajectory is committed to evolve toward 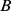. All we have to do is to sample the probability of observing the committed states with respect to all possible states. A useful way to express the transition function is to use the Heaviside function 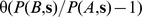 which is equal to one when 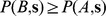 and zero otherwise. The total rate 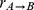 can the be measured by integrating Eq. (3) over all space of the sequences Next we write the expression of 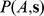 and 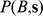 as the probability of observing a state with a sequence 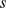 and configuration 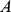 and 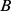 respectively
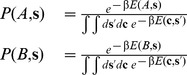
(5)where 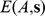 and 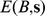 are the energies of structures 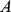 and 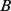 for sequence 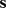. Here we used the Caterpillar energy field, sum of the terms in Eqs. 14 and 15. It is important to notice that the normalization is done over all possible sequences and structures. Note that this is different from either the probability of a sequence given a structure, or vice versa the probability of a structure given a sequence. The total rates can be then written combining Eq. (5) and Eq. (3) (see Information S1) and integrating over all sequences of 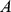 and 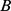 respectively.
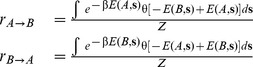
(6)Where 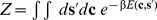 is the normalization constant and is the partition function of the 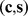 space. Eq. 6 cannot be directly computed because the integral 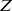 must be calculated measuring all possible sequences over all possible structures. On the other hand the rate constant 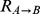 and 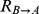 are obtained by dividing Eq. (6) by the probability of observing structure 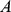 and structure 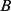 respectively
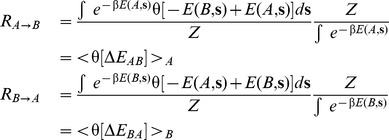
(7)where the brackets 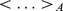 and 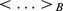 are ensemble averages, and indicate the average over the sequences weighted with the Boltzmann distribution. It is important to notice that if two structures are very different then we cannot guarantee that all the contributions to the average will come from sequences that fold either into 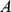 or 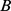. However, such sequences will have a lower Boltzmann weight compared to the sequences that fold into 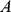 or 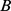. Hence, the number of non zero contributions will produce a small rate, equivalent to a forbidden jump.

### Rate Constant Calculation

We now have the final form of the rate constants, and it can be calculated by performing a Monte-Carlo simulation where we sample the sequences that have higher probability of being in 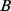 averaged in the ensemble of the sequences that fold into 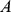. However the contribution of 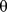 function is non zero only for sequences with an energy in 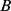 lower than in 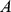, and because it is rare to have sequences with a low energy in both structures, the average will most frequently be close to zero. If instead we bias towards 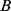, then
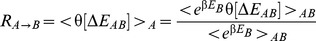
(8)where the ensemble average 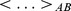 is performed with the product of the Boltzmann weights of 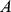 and 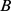 which results in an average over an effective system defined by the sum 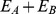, and 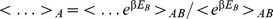. This is the natural ensemble to sample the sequences that contribute most to the average in Eq. (7) because the point at which the two distributions cross each other is in the overlapping region between 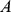 and 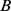. For this reason we will perform the average in Eq. (7) in the 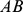 ensemble. Alternatively the sampling imposed by Eq. 8 can be interpreted as a simulation in the ensemble of sequences that fold into structure 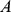 but in the presence of a bias towards sequences that fold into structure 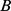. While the sampling goes back and forth in the joint ensemble of sequences that either fold in 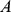 or 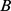 Eq. 8 computes the fraction of sequences that have a preference to fold in structure 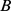. Now because we have reduced all the equations to quantities that we can calculate in the 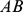 ensemble all we have to do is to perform a single simulation for each 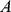, 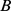 pair and compute the the rate from Eq. (8). In the supplementary material we derive the Metropolis acceptance rule for sampling the ensemble 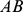. Each rate is then sampled by applying the design procedure described above to the joined 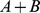 ensemble for each 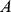, 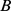 pair with the following acceptance rule
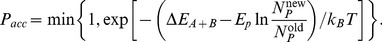
(9)Such an acceptance rule also guarantees that we do not include homopolymers sequences in the rate calculations that with their large enthalpic weight might significantly alter the results towards non-physical solutions.

### Folding

In order to characterize the equilibrium configuration of each sequence, we compute the free energy 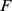 as a function of several order parameters. We will describe each case separately later in the manuscript, but they will be all similar to the following example: if 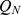 is an order parameter, then to compute 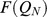, we used the following relation:
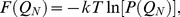
(10)where 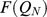 is the free energy of the state with order parameter 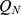 and 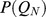 denotes a (normalized) histogram of the number of sampled conformations with order parameter 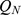. In practice, a direct (brute force) calculation of this histogram is not efficient, as the system tends to be trapped in local minima, especially at low temperatures. To solve this problem, we incorporated the Monte Carlo sampling approach of ref. [23] in the parallel-tempering algorithm of refs. [16], [24]. This scheme is very efficient in sampling both high and low free-energy states. A more detailed description of the algorithm can be found in Ref. [17].

## Results

### Folding and Design

Before calculating the the rates according to the Eqs. (8) we tested that the artificially generated structures used as stepping stones were designable and that the designed proteins could refold to the target structure. As a test of the complete set of stepping-stones was computationally unfeasible, we only considered three candidates (1PGB,2GYC-X and one of the generated stepping stones) from the list of stepping stones and tested the refolding properties of the sequences obtained with the design algorithm. Our approach was to take one of sequences generated for a target structure and then perform a Metropolis Monte-Carlo simulation in the configurational space of the protein chain. During the simulation we measured the root mean square distance (RMSD) (detailed description in the appendix) between the instantaneous configuration and the target native state. From the histogram (see Fig. S1 and [13] for 1PGB) of the observed values of RMSD we generated a free energy profile that demonstrated that the configurations closer to the native states (within 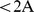 RMSD distance) were the most stable, hence we concluded that the protein was able to fold correctly. It is important to stress that in addition to the present test cases, the Caterpillar model correctly refolded all the 9 test proteins [13], out of which we used 8 designed and one natural sequence.

### Rates

**Figure 4:**
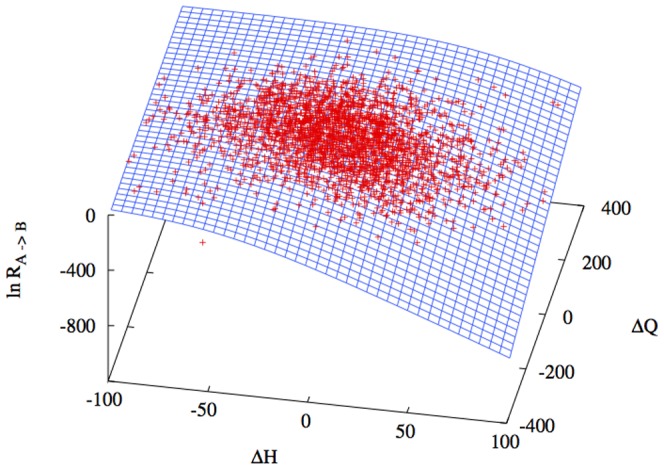
Plot of the logarithm of the rate constants 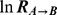
 **sampled according to** **Eq. 8** **as a function of the differences** 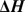 **and** 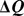 **of the Hydrogen bond energy and of the number of total contacts respectively.** The simulations data are fitted with the function 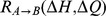 from Eq. 11. The points falls quite nicely on the surface indicating that the simple form of the rate in Eq. 11 captures the major trend of the simulation points. It is important to stress that for equally compact structures with the same number of hydrogen bonds the rate may still be influenced by important structural differences not included in this fit.

The next step in our study is to perform the design of the set of stepping stones. During the design simulation we sample the rate constants according to the Eqs. (8) using the Metropolis scheme described in the method section. Our objective is to correlate these rates with some variables measures that describe the structural difference between each pair of stepping stone. From the rates described by equation (7) it is evident that there is a strong dependence on the difference in energy between two structures for each sequence, hence in order to capture the fundamental structural differences between each stepping stone pair it is natural to select quantities such as the total hydrogen bond energy 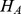 of structure 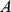 and 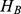 for structure 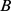, and the total number of residue contacts 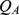 in structure 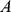 and the number 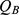 of contacts in 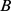. Another educated guess that we can make is that because the committor is a function only of the energy difference, we can expect the rate to behave similarly, this is also verified by distribution of the rates plotted as a function of the difference in the hydrogen bond energy 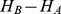 and the difference in the number of contacts 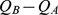 (Fig. (4)), if we remember that the plot is in log scale then the surface follows a step like function very similar to one that represents the committor function 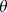. This indicates that the jumps follow an on/off transition process and also that we can extract a universal function that relates the rates with the difference in hydrogen bonds 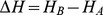 and in the number of contacts 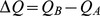 in the following way for the rates:
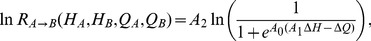
(11)where the values for 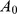, 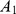, and 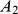 have been obtained by fitting to the simulation data. We have listed the final values for the parameters in Table 1. In Fig. (4) we plot the logarithm of the measured rates and the corresponding fitted rates surfaces from Eq. 11. The small errors over the parameter values of the plot show that there is good agreement between the predicted profile and the simulation data. This demonstrates the validity of our prediction of universal dependence of the rates on the structural variables.

**Table 1:** Values of the parameters in Eq. 11 and 12 used to fit the rates calculated with our simulations.

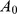	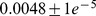
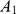	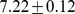
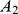	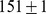
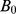	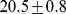
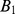	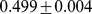
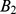	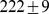

**Figure 5:**
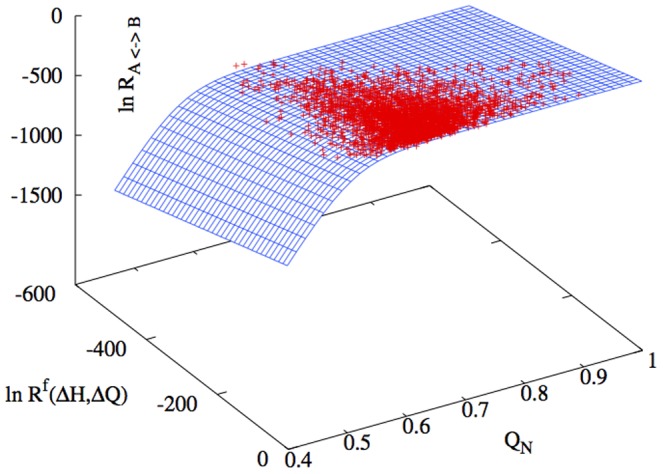
Rate constants plotted as a function of the number of common contacts 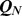 and the previously fitted expression of the rate constants 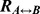 (see Eq. (11)). The profile shows a clear linear dependence of the rate constants from 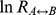 indicating that the assumption that it is sufficient to add a function 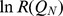 is reasonable. Although we do not have a wide range of values for 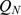 the data seems to fall on a sigmoidal function (eq. (12)) similarly to the fit in Fig. (11).

So far we have only considered the dependence of the rate constants on two structural parameters that cannot represent the total difference between two proteins. In particular we are missing information regarding how many contacts are in common between the pair of proteins. This information must play a role in the rate, as even for two proteins with the same number of hydrogen bonds and the same number of total contacts, we expect that the differences in the topology will make the population of sequences quite separate in energy. In other words if there are not many common contacts it is difficult to optimize two structures at the same time. A common measure of the similarity between two structures is the number of common native contacts 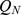. The dependence of the rate constants from 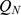 must not alter the detailed balance condition that we verified in the appendix (Section Detailed Balance). The condition of detailed balance requires that for each pair of stepping stones the ratio between the 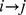 and 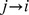 rate constants is equal to the ratio between the probabilities of observing the two structures, hence the ratio cannot depend on a quantity that cannot be factorized out. The simplest function is then the one resulting from the addition to the functions in Eq. (11) of a new term 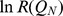. In order to maintain detailed balance, the new term must be symmetric under inversion of 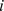 with 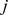. In order to determine the form of 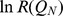 we plotted the rate constants in Fig. (5) as a function of the number of common contacts 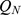 and the function 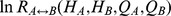 that describes the dependence of the rate constants as a function of the hydrogen bond energy difference and the difference in the total number of contacts. The plot shows that the data have a linear profile along 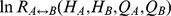, which supports our assumption for the factorization of the contribution of 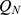. Hence we considered the following expression for 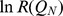:
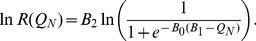
(12)where again the parameters 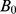 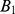 and 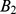 were obtained through a fit of the data in Fig. (5). The expression of 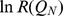 in eq. (12), is similar to the sigmoidal function used for 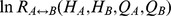. This is not a surprising result, considering the correct function must be close to the maximum rate 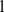 when the two proteins are very similar (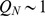).

**Figure 6:**
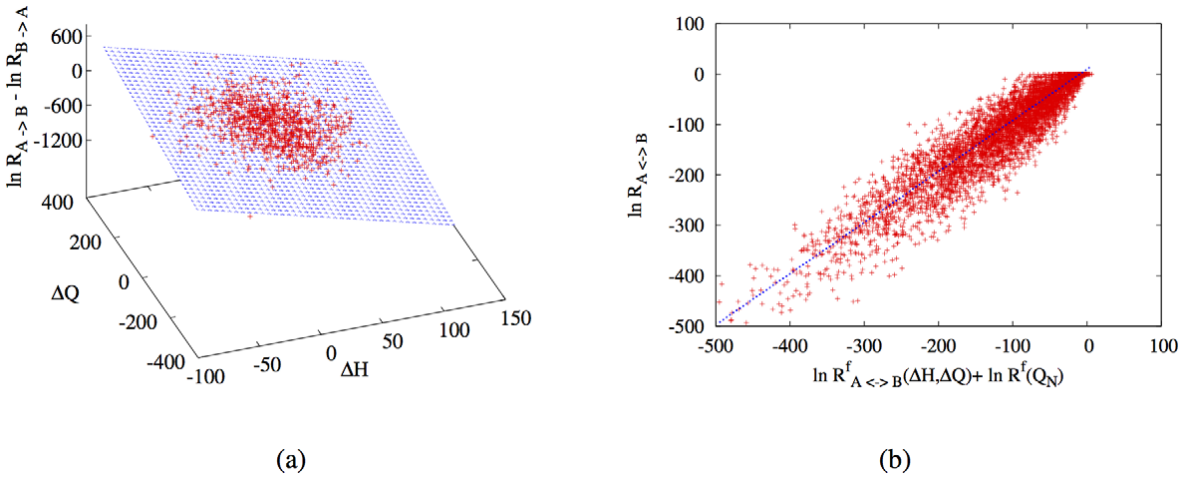
Consistency test for the fitted expression of the rate constants. (a) fit of the log of the ratio between the 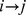 and 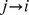 rate constants of the difference in the Hydrogen bond 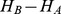 contribution and the number of total contacts 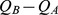 the perfect plane fit indicates that our expression and the data do verify the detailed balance condition. (b) Correlation plot between the final expression of the rate constants as a function of 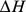 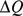 and 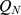 and the compute rates. The data follow a clear linear profile with a correlation coefficient of 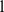.

An important property of the rate constants is that at equilibrium they must satisfy the system of equations characteristic of the underlying Markov model. In practice the following set of equations must be satisfied for each pair 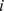 and 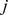


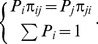
(13)where 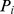 and 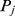 are the equilibrium probabilities. According to the expression of the rate constant that we fitted on the data (Eq. (11)) the ratio of the rate constants (or the difference between the logarithms) should give an expression that is factorisable into two functions that will depend only on the properties of 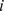 and 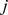. We can easily obtain the general solution to the system of equations (13) by expressing the probabilities 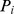 in the following form:
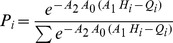
(14)in the appendix it is demonstrated explicitly that this form solves the system of equations in eq. (13). To further prove the validity of this construction we performed an independent fit of the logarithm of the ratio of the 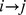 and 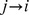 rate constants calculated for each 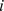 and 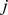. If our theory is correct the data should be optimally fitted by a plane 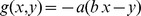 where 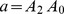, 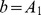, and 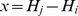 and 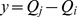. In Fig. (6) we plot the ratio fitted with the function 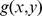, the points fall nicely on the plane and the optimized values of 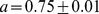 and 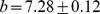 are equal to the expected values to within the experimental error.

Now that we have an analytic expression for the rate constants and the equilibrium probabilities we can consider a generalized system where we consider many more stepping stones than the one we used to explicitly compute the rate constants. This has two advantages: one is of course the minimal computational cost and the second is that it is now possible to solve the master equation of the evolutionary process that we modelled and extract the time dependent probability of reaching any state in the network with a given initial condition.

**Figure 7:**
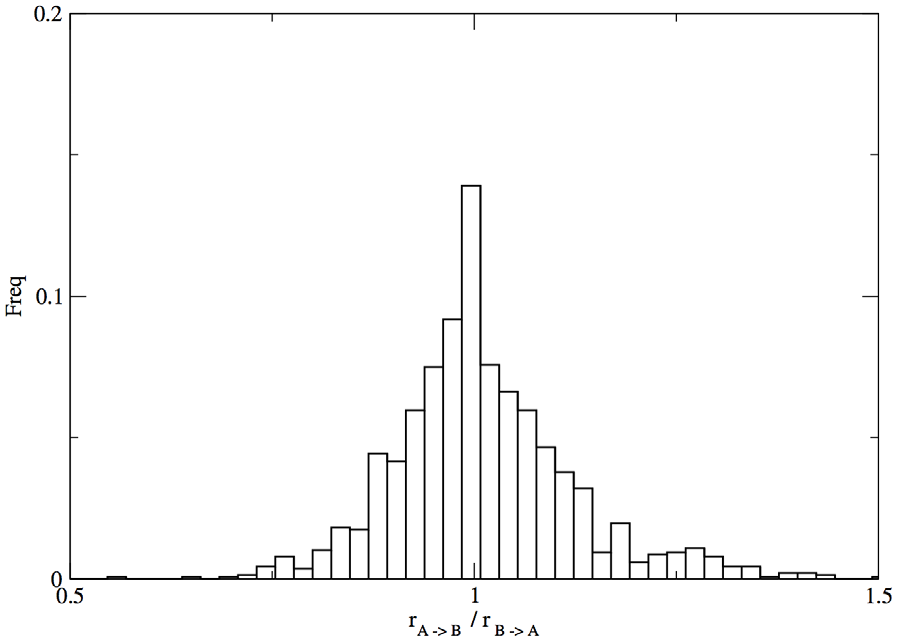
Distribution of the ratio between the the 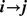 and 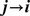 rates. The distribution is strongly peaked at one indicating that two rates are equal in the simulation error.

As we said in the methods section the rates from Eq. (6) cannot be calculated directly but we can instead obtained a measure relative to the the probability of observing one of the structures. If we consider as reference the probability of observing the reference structure A we can write
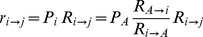
(15)where we used the fact that the 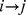 and 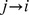 rate are equal (see Information S1 for formal proof), and 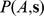 is the probability of observing structure A with all possible sequences. So the procedure consists in a simple rescale of the data obtained with the calculations of the rate constants between each stepping stone. In Fig. (7) we plot the ratio between the 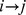 and 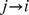 rates calculated using the expression in eq. (15), as expected the ratio is peaked at 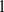 because of the detailed balance condition. Higher accuracy can be acquired with longer simulations to obtain better measures of the rate constants. However this precision is beyond the scope of this work.

## Discussion

In this work we have addressed the problem of understanding how the rate of appearance of novel protein structures depends on the various factors that constrain the evolutionary process. We have generalized the problem by modelling an evolutionary trajectory with a path that connects a set of structures. The ensemble of structures, or stepping stones, defines the degree of constraint under which the evolutionary process occurs. The total evolutionary rate is then determined by the rate of the jumps among the stepping stones. We then considered a test case in which we took two structures from the PDB and generated a large number of stepping stones between the two. From this set we measured the jumping rates between each pair of stepping stones. Finally, on the data we fitted the analytic dependence of the jump rate from simple structural difference between each stepping-stone, namely the difference in the number of hydrogen bonds and the number of inter-residues contacts (Eq. 11). In particular, this expression demonstrates that it is much easier to jump towards a compact structure with many hydrogen bonds than evolve towards a configuration that is either compact with few hydrogen bonds or non-compact with many hydrogen bonds. The simple form of the rate is a remarkable result, if one considers the complexity of the problem and the variety of structural differences between the stepping stones. Moreover, the expression for the rate respects the condition of detailed balance making it the perfect tool to define a Markovian process for evolution that can be numerically studied without the need of an expensive design of a large set of protein structures. A result that comes out naturally from our analysis is the probability of occurrence of a structure defined by Eq. 14, which can also be interpreted as the designability of a protein structure. We obtain the novel result that the designability of a protein does not depend just on how compact it is but, mainly on the optimization of both the number of hydrogen bonds and the number of contacts between the residues. Eq. 14 demonstrates that the higher the number of hydrogen bonds and residue contacts in a protein structure, the higher the probability of observing such a structure. However, because of the highly directional nature of the hydrogen bonds, is not always possible to increase at the same time the number of H-Bonds and the compactness. Hence, we predict that the protein configurational space does not have a single optimal structure but more probably an ensemble of equally designable structures. The results presented in this work have been obtained with a coarse grained model to represent the proteins. We chose the “Caterpillar" model because it allows for the design of realistic protein structures and does not require huge computational facilities. Of course, the model has limitations in offering a realistic representation of real proteins. In particular, we did not include explicit interaction with the solvent, and the accuracy in the refolding of real sequences still need extensive testing and improvement. However, we believe that the methodology here presented can be extended to any protein coarse-grained representation. Hence, an important extension of this work will be to consider more realistic models, provided that the resulting proteins are designable and the designed sequences refold to the respective the target structures.

## Supporting Information

Figure S1
**Free energies** 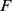 **(DRMSD)** 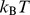 **of the designed sequences as a function of the root mean square distance (DRMSD) from their target structures for two test cases that we considered in this work: (a) the chain X of the 50S subunit of a secm-stalled E. Coli ribosome complex (PDB ID 2GYC) and (b) the model protein 172.** The free energy is shown for two temperatures, the first slightly below the folding temperature 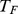 and the second above. At low temperatures, for all the target structures that we considered we found the minima of 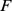 to be around 1.5 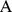 (corresponding to 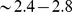 Å RMSD), indicating that the designed proteins are folded correctly on their targets.(TIFF)

Information S1 **In this appendix we derive the expression for he rates in** **Eqs. 6** **, we demonstrate that they obey detailed balance, and we show how to use a metropolis Monte Carlo method to measure the rates.** Finally we give details about the model and the biasing technique used to improve the sampling.
(PDF)
