# Supplementary figures and images for: Analytic Markovian Rates for Generalized Protein Structure Evolution
Source: PLoS One. 2012 May 23;7(5):e34228. doi: 10.1371/journal.pone.0034228 (PMC3367531; doi:10.1371/journal.pone.0034228)

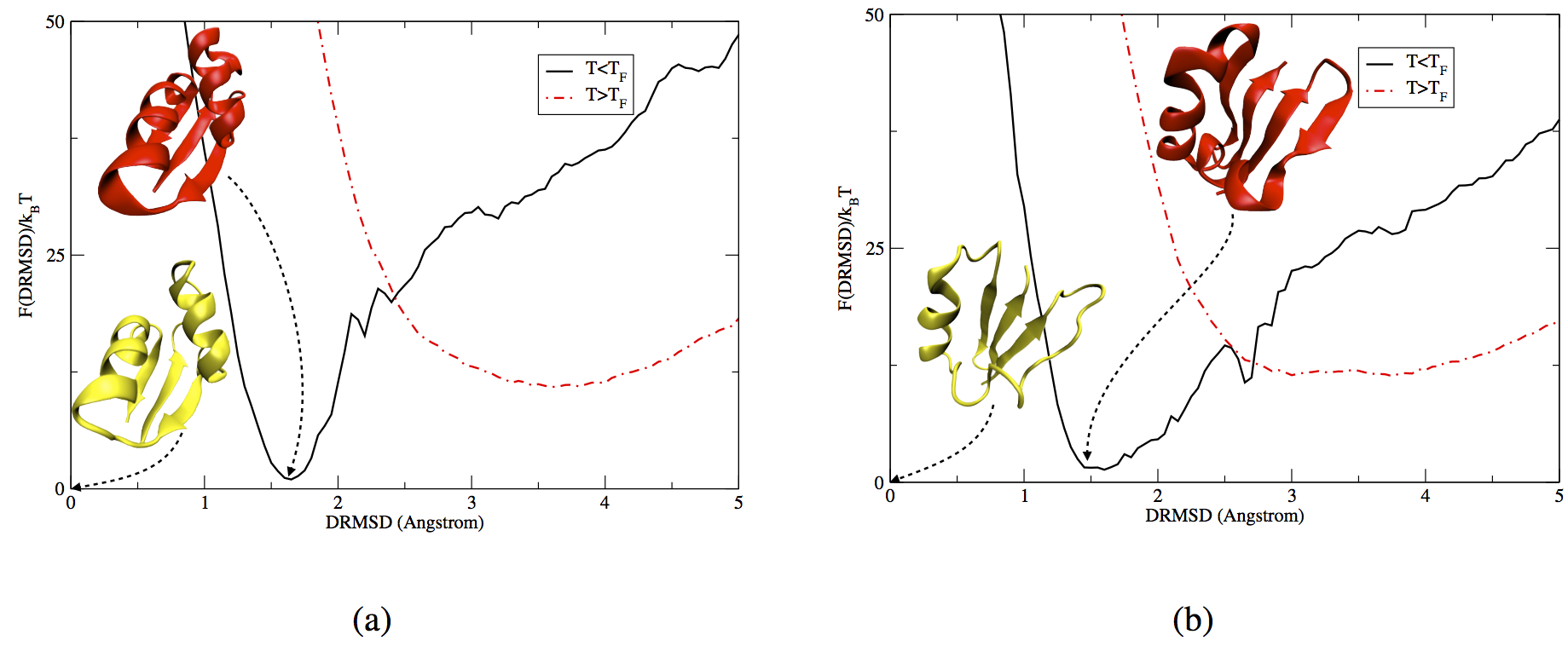

Supplement: Figure S1 — Free energies (DRMSD) of the designed sequences as a function of the root mean square distance (DRMSD) from their target structures for two test cases that we considered in this work: (a) the chain X of the 50S subunit of a secm-stalled E. Coli ribosome complex (PDB ID 2GYC) and (b) the model protein 172. The free energy is shown for two temperatures, the first slightly below the folding temperature and the second above. At low temperatures, for all the target structures that we considered we found the minima of to be around 1.5 (corresponding to Å RMSD), indicating that the designed proteins are folded correctly on their targets. (TIFF) [file pone.0034228.s001.tiff]
