# Supplementary material for: Analytic Markovian Rates for Generalized Protein Structure Evolution
Source: PLoS One. 2012 May 23;7(5):e34228. doi: 10.1371/journal.pone.0034228 (PMC3367531; doi:10.1371/journal.pone.0034228)
Supplement: Information S1 — In this appendix we derive the expression for he rates in Eqs. 6 , we demonstrate that they obey detailed balance, and we show how to use a metropolis Monte Carlo method to measure the rates. Finally we give details about the model and the biasing technique used to improve the sampling. (PDF) [file pone.0034228.s002.pdf]

SUPPORTING INFORMATION S1

Rate theory

In this section we will show more explicitly the calculations to obtain the expression for the rates in Eqs. 6. We start by substituting Eq. (5) into the expression in Eq. (3) and the equivalent for B we obtain

$$r_{A\to B} = \frac{\int e^{-\beta E(A,\mathbf{s})}\theta\left[\frac{P(B,\mathbf{s})}{P(A,\mathbf{s})}-1\right]d\mathbf{s}}{\int\int d\mathbf{s}d\mathbf{c}e^{-\beta E(\mathbf{c},\mathbf{s})}} \tag{S1}$$

$$r_{B\to A} = \frac{\int e^{-\beta E(B,\mathbf{s})}\theta\left[\frac{P(A,\mathbf{s})}{P(B,\mathbf{s})}-1\right]d\mathbf{s}}{\int\int d\mathbf{s}d\mathbf{c}e^{-\beta E(\mathbf{c},\mathbf{s})}} \tag{S2}$$

which can be rewritten by further substituting the expression of the probabilities $P(A,\mathbf{s})$ and $P(B,\mathbf{s})$ from Eq. (5), where we have substituted the sum with an integral over the sequence space.

$$r_{A\to B} = \frac{\int e^{-\beta E(A,\mathbf{s})}\theta\left[e^{-\beta[E(B,\mathbf{s})-E(A,\mathbf{s})]}-1\right]d\mathbf{s}}{\int\int d\mathbf{s}d\mathbf{c}e^{-\beta E(\mathbf{c},\mathbf{s})}} = \frac{\int e^{-\beta E(A,\mathbf{s})}\theta\left[-E(B,\mathbf{s})+E(A,\mathbf{s})\right]d\mathbf{s}}{Z} \tag{S3}$$

$$r_{B\to A} = \frac{\int e^{-\beta E(B,\mathbf{s})}\theta\left[e^{-\beta[E(A,\mathbf{s})-E(B,\mathbf{s})]}-1\right]d\mathbf{s}}{\int\int d\mathbf{s}d\mathbf{c}e^{-\beta E(\mathbf{c},\mathbf{s})}} = \frac{\int e^{-\beta E(B,\mathbf{s})}\theta\left[-E(A,\mathbf{s})+E(B,\mathbf{s})\right]d\mathbf{s}}{Z} \tag{S4}$$

Detail Balance

At this stage we can check that we obey detailed balance to do that we will use an approximate form for the Heaviside function that is particularly good for large β or small temperatures

$$\theta(\beta x) \approx \frac{1}{1+e^{-\beta x}} \tag{S5}$$

If we substitute Eq. (S5) into Eq. (S3)

$$r_{A\to B} = \frac{\int e^{-\beta E(A,\mathbf{s})}\frac{1}{1+e^{[\beta(E(B,\mathbf{s})-E(A,\mathbf{s}))]}}d\mathbf{s}}{Z} \tag{S6}$$

$$r_{B\to A} = \frac{\int e^{-\beta E(B,\mathbf{s})}\frac{1}{1+e^{[\beta(E(A,\mathbf{s})-E(B,\mathbf{s}))]}}d\mathbf{s}}{Z} \tag{S7}$$

if we rearrange the exponentials

$$r_{A\to B} = \frac{\int e^{-\beta E(A,\mathbf{s})}e^{-\beta E(B,\mathbf{s})}\frac{1}{e^{-\beta E(B,\mathbf{s})}+e^{-\beta E(A,\mathbf{s})}}d\mathbf{s}}{Z} \tag{S8}$$

$$r_{B\to A} = \frac{\int e^{-\beta E(B,\mathbf{s})}e^{-\beta E(A,\mathbf{s})}\frac{1}{e^{-\beta E(A,\mathbf{s})}+e^{-\beta E(B,\mathbf{s})}}d\mathbf{s}}{Z} \tag{S9}$$

It is easy to see that Eq. (S8) and Eq. (S9) are identical and hence detailed balance is respected.

Metropolis acceptance

If we want to sample the distribution $P(A)P(B)$ then we can use the Metropolis method. We start from the detailed balance equation:

$$P(Old)\Pi(Old \to New) = P(New)\Pi(New \to Old) \tag{S10}$$

where $P(Old) = P_A(O)P_B(O)$ and $P(New) = P_A(N)P_B(N)$

$$\frac{\Pi(Old \to New)}{\Pi(New \to Old)} = \frac{P_A(N)P_B(N)}{P_A(O)P_B(O)} \tag{S11}$$

If we now remember that P_A and P_B are Boltzmann distributed then we can calculate the transition probabilities and hence our acceptance rule

$$\frac{\Pi(Old \to New)}{\Pi(New \to Old)} = \frac{e^{-\beta E_A(N)}/Q_A e^{-\beta E_B(N)}/Q_B}{e^{-\beta E_A(O)}/Q_A e^{-\beta E_B(O)}/Q_B} \tag{S12}$$

So if we group the exponents together we get that the final acceptance rule depends on the variation of the sum of Hamiltonians at every step.

$$\frac{\Pi(Old \to New)}{\Pi(New \to Old)} = \frac{e^{-\beta E_{All}(N)}}{e^{-\beta E_{All}(O)}} = e^{-\beta \Delta E_{All}}. \tag{S13}$$

Coarse-grained Monte Carlo simulations

The caterpillar model [13] is a 5-bead model with the C_α augmented by the full main atomic positions to introduce directional hydrogen bonds. The degrees of freedom of the model are the torsional angles ϕ and ψ; all other structural parameters are kept fixed at values from the literature [1]. The C, O, N, H positions were determined from the C_α (see Fig.1 in Ref [13]). The side chain interactions are represented by and effective C_α-C_α sphere-sphere interaction energy given by

$$E_{ij}(r_{ij}) = \alpha\, \varepsilon_{ij} \left[1 - \frac{1}{1.0 + \mathrm{e}^{2.5[1/\text{Å}]\left(r_{\max} - r_{ij}\right)}} \right] \tag{S14}$$

where r_{ij} is the distance between the C_α atoms at the centers of spheres i and j and $r_{\max}$ ($r_{\max} = 12\,\text{Å}$) is the distance at which $E_{ij} = \varepsilon_{ij}/2$; $\alpha = 1/4$ is a scale factor. This expression provides a continuous square well form for the sphere-sphere interaction energy. To determine the parameter ε_{ij} we made use of the model of Betancourt and Thirumalai (BT) [2], in which the interaction energies were derived from a calculation of the contact frequency in the PDB. This potential had been used primarily for lattice proteins, but it is

also appropriate for the caterpillar model, which employs a square-well-like potential. Backbone hydrogen bonds were modeled with a 10-12 Lennard-Jones type potential using the expression [3]

$$E_H = -\varepsilon_H \left(\cos\theta_1 \cos\theta_2\right)^{\nu} \left[5\left(\frac{\sigma}{r_{\mathrm{OH}}}\right)^{12} - 6\left(\frac{\sigma}{r_{\mathrm{OH}}}\right)^{10}\right], \tag{S15}$$

where r_{OH} is the distance between the hydrogen atom of the amide group (NH) and the oxygen atom of the carboxyl group (CO) of the main chain. We set $\sigma = 2.0$ Å, $\varepsilon_H = -3.1\ k_B T$, and $\nu = 2$; the values are given in [3].

VIRTUAL-MOVE PARALLEL TEMPERING

The VMPT scheme is a combination of the adaptive parallel tempering algorithm [4] and the waste recycling method developed by Frenkel [23]. This method consists in using the information about the reject moves to generate a bias potential that helps the system visit regions of the phase space that are otherwise difficult to reach. In the conventional MCMC method all information about rejected trial moves is discarded. Recently Frenkel has proposed a scheme that makes it possible to include the contributions of rejected configurations in the sampling of averages [23]. In the standard parallel tempering (PT) scheme, we only retain information about PT moves that have been accepted. However, in the spirit of Ref. [23], we can include the contribution of all PT trial moves, irrespective of whether they are accepted. The weight of the contribution of such a virtual move is directly related to its acceptance probability. For instance, if we use the symmetric acceptance rule for MC trial moves, then the weights of the original and new (trial) state in the sampling of virtual moves are given by

$$\begin{aligned} P_N &= \frac{e^{\Delta\beta\Delta E_{O\to N} + \Delta W_{O\to N}}}{1 + e^{\Delta\beta\Delta E_{O\to N} + \Delta W_{O\to N}}} \\ P_O &= \frac{1}{1 + e^{\Delta\beta\Delta E_{O\to N} + \Delta W_{O\to N}}}, \end{aligned}$$

where W is a bias potential. We are not limited to a single trial swap of state i with a given state j. Rather, we can include all possible trial swaps between the temperature state i and all $N-1$ remaining temperatures. Our estimate for the contribution to the probability distribution P_i corresponding to temperature i is then given by the following sum

$$\begin{aligned} P_i(Q) = &\sum_{j=1}^{N-1} \left(\frac{1}{1 + e^{\Delta\beta_{ij}\Delta E_{ij} + \Delta W_{ij}}}\right) \delta(Q_i - Q) + \\ &\sum_{j=1}^{N-1} \left(\frac{e^{\Delta\beta_{ij}\Delta E_{ij} + \Delta W_{ij}}}{1 + e^{\Delta\beta_{ij}\Delta E_{ij} + \Delta W_{ij}}}\right) \delta(Q_j - Q), \end{aligned}$$

where the delta functions select the configurations with order parameter Q. The combination with the parallel tempering is particularly efficiently because the information about the sampled states is shared between all the simulations running at different temperatures, that naturally will tend to explore different regions of the phase space. In the case of proteins it means that at low temperature it is possible to know what bias is needed to reach the unfolded state because that is sampled more often at higher temperatures.

REFERENCES

[1] Creighton (1992) Proteins. W.H.Freeman & Co Ltd 2nd Revised edition.

[2] Betancourt M, Thirumalai D (1999) Pair potentials for protein folding: Choice of reference states and sensitivity of predicted native states to variations in the interaction schemes. Protein Science 8: 361369.

[3] Irbäck A, Sjunnesson F, Wallin S (2000) Three-helix-bundle protein in a ramachandran model. P Natl Acad Sci USA 97: 13614-13618.

[4] Faller R, Yan Q, de Pablo J (2002) Multicanonical parallel tempering. J Chem Phys 116: 54195423.
